# Microstructure, Texture, Electrical and Mechanical Properties of AA-6063 Processed by Multi Directional Forging

**DOI:** 10.3390/ma11122419

**Published:** 2018-11-29

**Authors:** Alireza Dashti, Mohammad Hossein Shaeri, Reza Taghiabadi, Faramarz Djavanroodi, Farzaneh Vali Ghazvini, Hamid Javadi

**Affiliations:** 1 Department of Materials Science and Engineering, Imam Khomeini International University (IKIU), Qazvin 3414916818, Iran; alirezad1991@yahoo.com (A.D.); taghiabadi@eng.ikiu.ac.ir (R.T.); 2 Mechanical Engineering Department, Prince Mohammad Bin Fahd University, Al Khobar 31952, Saudi Arabia; f.djavanroodi@imperial.ac.uk; 3 Department of Mechanical Engineering, Imperial Collage London, London SW7, UK; 4 Ecole de Technologie Supérieure, Department of Mechanical Engineering, Montréal, QC H3C 1K3, Canada; farzaneh.vali-ghazvini@ens.etsmtl.ca (F.V.G.); hamid.javadi@ens.etsmtl.ca (H.J.)

**Keywords:** MDF process, AA-6063 aluminum alloy, microstructure, texture, mechanical properties, electrical properties

## Abstract

In current research, the effect of the multi-directional forging (MDF) process on the microstructure, texture, mechanical and electrical properties of AA-6063 under different heat treatment conditions at various MDF temperatures was studied. The annealed AA-6063 alloy was processed up to three passes of MDF at ambient temperature. Three passes of this process were also applied to the solution-treated AA-6063 at ambient temperature and 177 °C. Microstructural investigations demonstrated that the MDF process led to a significant reduction in the average grain size as well as a considerable increase in the fraction of low angle grain boundaries. Texture analysis revealed that copper and Goss textures were mainly developed within the annealed and solution-treated samples of AA-6063, respectively. The hardness and shear strength values of all processed samples also showed a sizeable improvement compared to the initial heat-treated samples. For example, the hardness and shear yield strength value of the solution-treated sample MDFed for three passes showed more than 100 and 70% increase, respectively. The effect of the MDF process on the electrical conductivity of AA-6063 under different heat treatment conditions at various temperatures was negligible. So, it can be concluded that the MDF process increased the mechanical properties without an appreciable decrease in electrical conductivity.

## 1. Introduction

Severe Plastic Deformation (SPD) has proved to be a reliable method for producing ultrafine-grained (UFG) metals and alloys. Multi-directional forging (MDF) as one of the most attractive SPD processes, was first applied in the 1990s to develop UFG structure in bulk materials. Despite the lower strain homogeneity achieved by MDF compared with other common SPD processes like equal channel angular pressing (ECAP) and high-pressure torsion (HPT), this method is well-suited to generating a UFG structure in rather brittle materials considering its relatively low specific load and the possibility to conduct the process at elevated temperatures. Other advantages of MDF include its high efficiency and low processing cost. Choosing appropriate temperatures and strain rates can lead to a desired nanocrystalline structure even for large billets [1,2,3,4,5].

AA-6063 is one of the most commonly used Al alloys in the heat treatable 6xxx series. This moderate-strength alloy is highly extrudable and shows excellent weldability, brazeability, and fair machinability as well as a prominent resistance to all types of corrosion. Because of these outstanding properties, the alloy has found a varied range of applications including architectural extrusions, pipe, truck and trailer flooring, furniture, road transport, rail transport, doors, windows, and irrigation pipes [6]. Considering the numerous applications of this widely used alloy which mostly require proper mechanical characteristics, it is interesting to produce it with a UFG microstructure by SPD processes.

It is well known that heat-treatable aluminum-based alloys such as the 6xxx and 7xxx series can be strengthened appreciably by precipitation hardening. In addition, grain refinement and strain hardening during the SPD process can deliver a significant improvement to the alloy’s strength [2,7,8]. Previous reports revealed that SPD processes, such as ECAP, caused a considerable improvement in the mechanical properties of 6xxx series alloys [9,10,11,12,13]. For instance, the refined and homogeneous microstructure can be achieved during ECAP process of the AA-6063 alloy at an optimum die channel angle of 90°. The process also led to a significant increase in the ultimate compressive strength, yield strength, compression modulus, and microhardness of this alloy compared to the as-received material [9]. In another research, a combination of pre-ECAP solid-solution with the post-ECAP aging treatment of AA-6061 was demonstrated to be more effective at strengthening the alloy than pre-ECAP peak-aging [10,11]. AA-6061 samples processed by multi-directional forging also exhibited high strength and high ductility. Processing this alloy subjected to prior solutionizing treatment through MDF at cryogenic temperature yielded a homogeneous microstructure with ultrafine grain morphology. 6061 aluminum alloy was also subjected to the MDF process at liquid nitrogen temperature that led to an improvement in hardness and tensile strength as a result of equiaxed sub-grain structure formation and the presence of high dislocation density [12,13].

A few investigations have been carried out on multi-directional forging of 6xxx aluminum alloys, but AA-6063 has not been among relevant researches. There are multiple points that make this research worth studying. First, the researches on the texture and specifically electrical properties of aluminum alloys processed by SPD processes are very limited. Second, the effect of the MDF process in particular on the electrical properties and texture of AA-6063 has not been studied before at all. Considering the fact that AA-6063 alloy has some applications in electrical devices, such as electrical components, conduits, and conductors, that require reasonable conductivity as well as other lightweight and strength demanding applications including the finer details of aircrafts and aerospaces and also extreme sports equipment, knowing the impact of the MDF process on the microstructure, mechanical, and electrical properties and texture development of the alloy can be very interesting. It is worth mentioning that the MDF process is much simpler and cheaper compared with other SPD processes in many cases. Therefore, due to the low alloying elements of AA-6063, by applying the MDF process as a cheap and simple SPD process, a high-strength AA-6063 alloy with reasonable electrical conductivity could be fabricated. The fabricated alloy can replace some high-strength 6xxx aluminum alloys containing higher alloying elements [14,15,16].

The present investigation was intended to examine the effect of the MDF process on the microstructure, texture, and electrical and mechanical properties of AA-6063 in the annealed and solution-treated states at various temperatures and pass numbers. The core objective was to study grain structure and how it affects the above-mentioned properties. Therefore, electron back scatter diffraction (EBSD) was employed to investigate the evolution of grain structure and texture. In addition, shear strength and microhardness tests, as well as the eddy current method, were used to measure the mechanical and electrical properties.

## 2. Materials and Methods

The AA-6063 used in this study has a nominal composition of (as follows:) Al-0.5 Mg-0.39 Si-0.17 Fe-0.01 V-0.01 Ga (wt. %). Rectangular parallelepiped samples with dimensions of 15 × 10 × 10 mm^3^ were machined from the as-received cast billet. Before MDF, the specimens were subjected to 2 different heat treatments to produce different initial metallurgical states. The annealed samples (AS) were produced via a 2-h heat treatment at 415 °C (furnace cooling). The solid solution-treated samples (SS) were solution heat treated at 520 °C for 2 h and water quenched to form a super saturated solid solution (SSSS). The solution-treated samples were immediately transferred to a −15 °C freezer to prevent the aging process at room temperature (RT). The samples were then processed by MDF for up to 3 passes. The annealed samples were only processed at room temperature, while the solution-treated samples were process at room and 177 °C temperatures. To study the dynamic aging during MDF, 177 °C was selected for a high-temperature MDF process as this temperature is the T6 heat treatment aging temperature (solution treatment at 520 °C + water quenching + peak aging at 177 °C) of AA-6063 alloy [6].

The MDF die was made of H13 tool steel, and its central cavity has dimensions of 40 × 15 × 10 mm^3^. The MDF die and the related tooling used in this process were presented in our previous work [5]. A 100-ton hydraulic press with 0.5 s^−1^ strain rate was used to strain the samples by 0.5. The imposed strain in each pass was verified and calculated as follows [17]:

(1) $$\varepsilon =\frac{2}{\sqrt{3}}\ln\left(\frac{H}{W}\right)$$

where *H* and *W* are the height and width of the sample, respectively [17].

**Figure 1:**
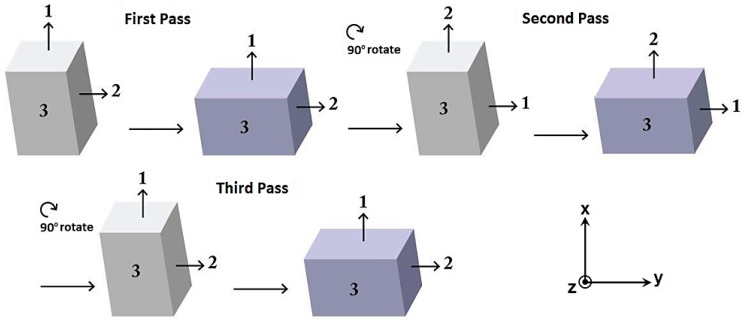
Schematic view of the multidirectional forging (MDF) process applied in current research.

As shown in Figure 1, the samples were rotated by 90° around the direction perpendicular to the side 3 after each pass of the process [17]. They were pressed up to a maximum of 3 passes because cracking and segmentation were observed to occur during the fourth pass of MDF in the solution-treated specimens. Graphite-based lubricant “Moly Coat 1000 Paste” was used for the lubrication of the die and samples.

EBSD analyses were performed on a field emission scanning electron microscope (FE-SEM, Hitachi high technologies corporation, Tokyo, Japan) HITACHI SU-70 operating at an accelerating voltage of 15 kV. EBSD scans were acquired via HKL CHANNEL5 (Oxford Instruments, Hobro, Denmark). The size and resolution of the maps vary from one process condition to another depending on the size of the grains. Before EBSD characterization, the samples were cut in half along their length, and the central square cross-sections were manually polished down to 1 µm via diamond polishing followed by mechano-chemical polishing with a 0.05 µm silica colloidal solution using a BUEHLER Vibrometer (Buehler Company, Lake Bluff, IL, USA) for 24 h.

Shear punch and microhardness tests were carried out to document the mechanical properties of the samples. Vickers microhardness measurements with 0.5 kg force and a dwell time of 15 s were performed according to the standard ASTM E-384. The hardness of the middle square cross-section of the samples along their length was measured at least five times, and the average values are reported. Shear punch testing (SPT) is a suitable test to measure the mechanical properties of small-sized samples [5,17,18]. Square Sheets with a thickness of 0.8 mm were cut from the middle of the samples along their length. The sheets were polished down to a thickness of 0.7 mm and punched using a shear punch fixture with a 6.2 mm diameter flat cylindrical punch and a 6.25 mm diameter receiving hole. All the shear punch tests were performed at the room temperature using a Zwick/Roell Z100 Universal Testing machine (ZwickRoell GmbH & Co., Ulm, Germany). The experiments were conducted at a constant cross-head speed of 10–3 mm/s^−1^. The shear test was repeated at least 3 times for each sample and the average values are reported. The schematic of equipment used for the shear punch test is illustrated in our previous studies [5,18]. After obtaining the load displacement curves in this test, the shear stress was calculated as follows [18,19,20,21]:

(2) $$\tau =\frac{P}{2\pi r_{ave}t}$$

where *τ*, *P*, and *t* represent the shear stress, applied force and specimen thickness, respectively and *r_avg_* is obtained using the following relation:

(3) $$r_{ave}=\frac{r_{punch}+r_{die}}{2}$$

SPT curves were obtained by plotting shear strength vs. normalized punch displacement. The following equation was used for measuring normalized punch displacement [18,19,20,21]:

(4) $$d=\frac{h}{t}$$

A SIGMOR 100 conductivity meter (New Chapel Electronics, Fairford, UK) was also used to measure the electrical conductivity changes using the eddy current method according to standard ASTM E1004. The electrical conductivity results are reported as the percentage of conductivity relative to the International Annealed Copper Standard (% IACS). The conductivity measurements were performed at room temperature. All measurements were repeated 3 times, and the average value is reported for each one.

## 3. Results and Discussion

### 3.1. Microstructure

**Figure 2:**
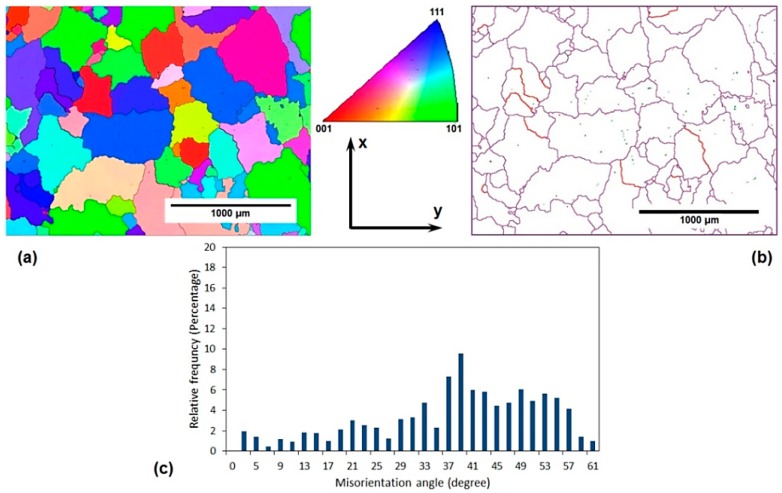
Microstructure features of initial annealed AA-6063 alloy before MFD process: (**a**) the electron back scatter diffraction (EBSD) grain orientation map, (**b**) the EBSD grain boundary map, and (**c**) the misorientation angle distribution diagram.

**Figure 3:**
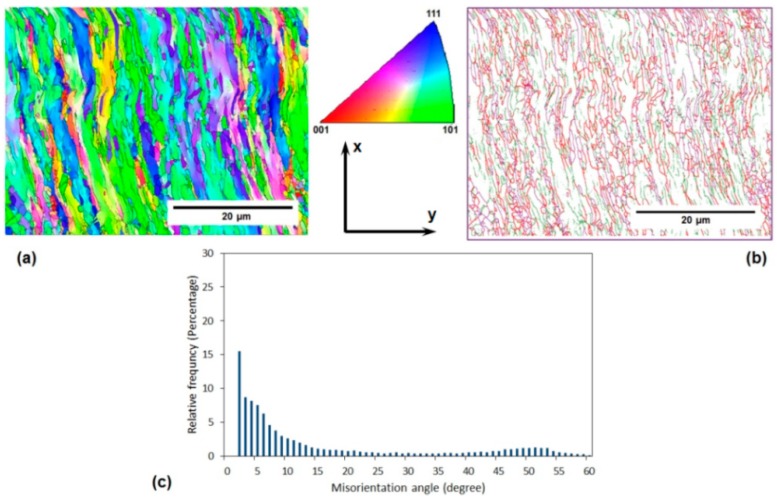
Microstructure features of the annealed AA-6063 alloy after three passes of MFD process at room temperature (RT): (**a**) the EBSD grain orientation map (IPF map) according to direction 2, (**b**) the EBSD grain boundary map, and (**c**) the misorientation angle distribution diagram.

Figure 2 and Figure 3 depict some microstructural aspects of the annealed AA-6063 alloy before and after three passes of the MDF process, respectively. These aspects include EBSD grain orientation maps, related grain boundary maps, and misorientation angle distribution. The unit triangle which determined the grain colors by the orientation of each grain is also depicted in Figure 2. The unit triangle is the same for all grain color maps. The EBSD analysis results showed that the average grain size of the annealed samples decreased from about 150 µm to less than 800 nm after being processed for up to three passes of MDF. The area fraction method was applied to measure the average grain size and the grain boundaries with a misorientation angle of more than 15° considered as the effective grain boundaries. It also clear that most of the grains are elongated in the direction of shear bands after the MDF process.

The grain boundary maps in Figure 2b and Figure 3b show the upsurge of low angle grain boundaries (LAGBs) in the processed sample where magenta, green, and red lines represent the boundaries with the misorientation angle of higher than 15°, 5–15°, and 2–5°, respectively. The fraction of LAGBs after three passes represented more than 65% compared to only 9% for the initial material. Similarly, a significant increase in the surface density of high angle grain boundaries (HAGBs) can be observed when comparing Figure 2b with Figure 3b. It can be deduced that the surface density of HAGBs and LAGBs increases considerably after three passes of MDF.

The microstructural developments in metals and alloys during the SPD process is related to the evolution of the dislocation substructures. The density of dislocations increases significantly with the applying strain at the initial stages of deformation, leading to the development of a cellular substructure. As the applied strain increases, the dislocation cells turn into cell blocks, and dislocation sub-boundaries are formed within these blocks generating LAGBs [22,23,24]. A variety of deformation bands including micro-shear bands (MSBs), start to appear at medium strains, resulting in the fragmentation of original grains. Finally, the LAGBs transform into HAGBs, and an ultrafine-grained structure progressively develops [25,26,27]. Previous studies of SPD processes also confirm the replacement of cells and subgrains formed at relatively lower strains by ultrafine grains as the gradual increase of dislocation density in the cell walls transform them into an ultrafine-grained structure [27,28,29,30].

The macrostructure of the samples processed by MDF will show an X-shaped pattern across their rectangular cross-section (the plane perpendicular to the direction z in Figure 1) as a result of MSBs accumulation as presented by Moghaddam et al. [31]. MSBs can contribute to the formation of sub-boundaries within the HAGBs [27,31]. EBSD images were taken from the central square cross-section of the samples along their length (the plane perpendicular to the direction y in Figure 1), where the accumulated MSBs would appear like a ribbon (considering the abovementioned X-like pattern) close to the center of the specimens’ cross-section. The obtained results including grain refinement and the significant increase of LAGBs can be attributed to the accumulation of MSBs that also leads to the elongation of the grains along these deformation bands mostly in the central area of the aforementioned cross-section.

The lower imposed strain in each pass of the MDF process compared to the other common SPD processes such as ECAP can be considered as the main reason of high fraction of LAGBs in the MDFed sample. As a result of a relatively low imposed strain, accumulated strain after three passes of MDF (ε = 1.5) is presumably inadequate to transform LAGBs into HAGBs [13,32].

**Figure 4:**
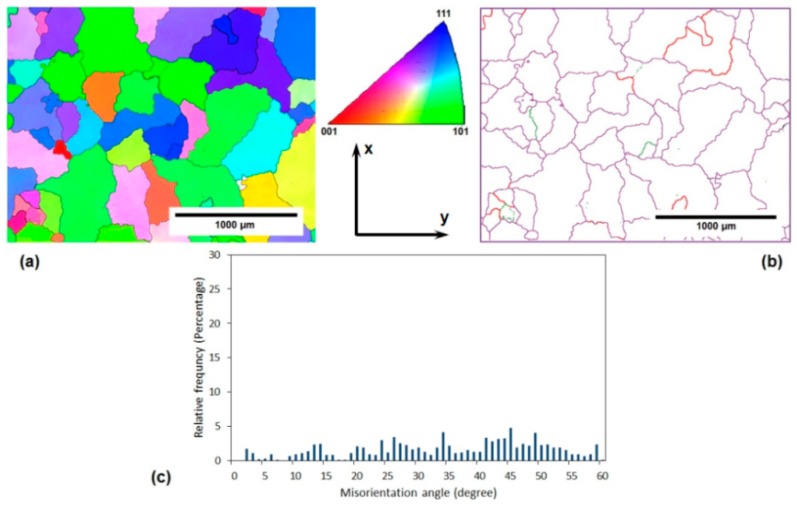
Microstructure features of initial solution-treated AA-6063 alloy before MFD process: (**a**) the EBSD grain orientation map, (**b**) the EBSD grain boundary map, and (**c**) the misorientation angle distribution diagram.

**Figure 5:**
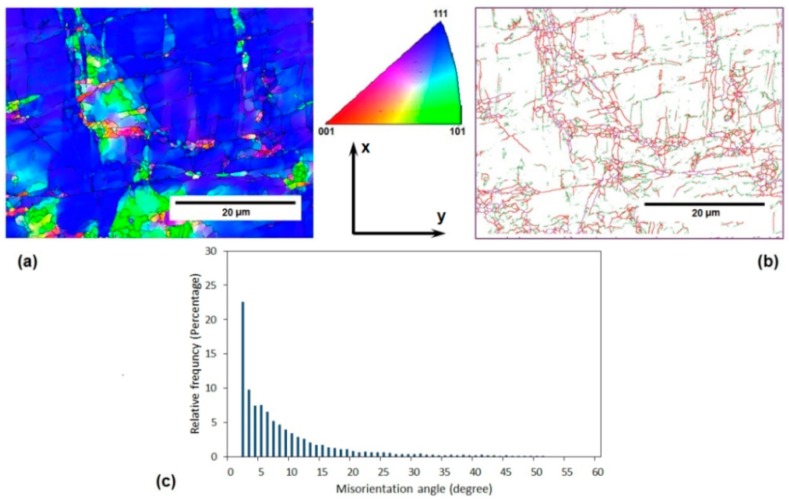
Microstructure features of solution-treated AA-6063 alloy after three passes of MFD process at RT: (**a**) the EBSD grain orientation map, (**b**) the EBSD grain boundary map, and (**c**) the misorientation angle distribution diagram.

**Figure 6:**
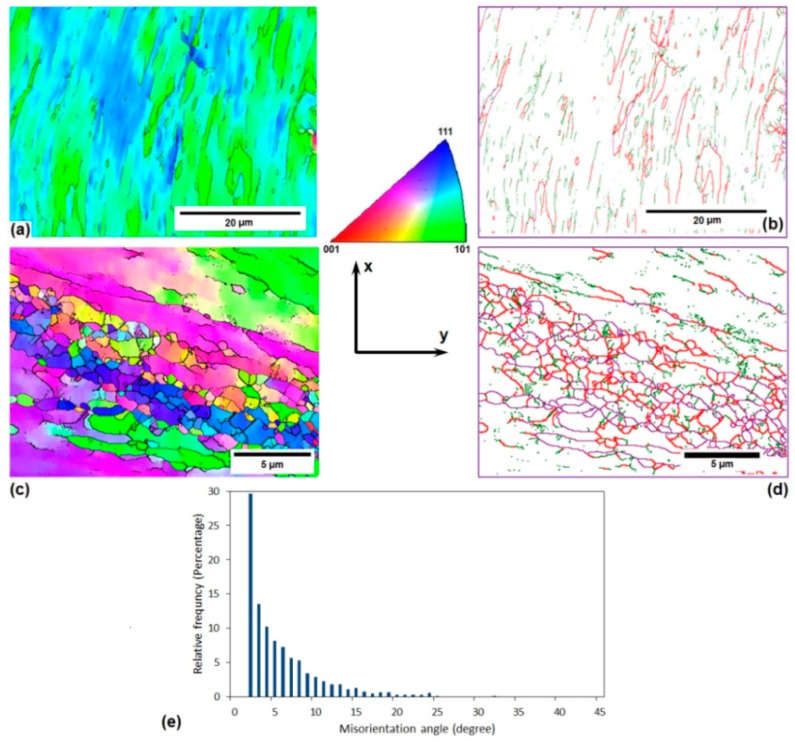
Microstructure features of solution-treated AA-6063 alloy after three passes of MFD process at 177 °C: (**a**,**c**) the EBSD grain orientation map, (**b**,**d**) the EBSD grain boundary map, and (**e**) the misorientation angle distribution diagram.

The microstructure features of the solution-treated AA-6063 alloy before and after three passes of the MDF process at both room temperature and 177 °C are demonstrated in Figure 4, Figure 5 and Figure 6. Two different EBSD magnifications were used to confirm the formation of very small grains in some regions of MDFed sample at 177 °C. The EBSD analysis results of the solution-treated samples showed that after three passes of MDF at RT and 177 °C, the average grain size of the initial samples has been reduced from 200 µm to 1000 and 1500 nm, respectively. The grain boundary map of the MDFed samples at both temperatures indicate a considerable increase in the LAGBs and HAGBs density that can be justified according to the previously mentioned analyses for the annealed samples. As can be seen in the misorientation angle distribution diagrams, more than 80% of the grain boundaries are LAGBs in the MDFed samples at both temperatures, while only 14% of them are LAGBs in the initial sample. The results including grain refinement and a significant increment in the fraction of LAGBs and grain elongation can be ascribed to the accumulation of MSBs which is similar in the annealed samples. It should be pointed out that the very fine equiaxed grains were formed after three passes of MDF at the center of the specimens’ cross-section as a result of the high imposed strain and accumulated MSBs in those regions (Figure 6b,d).

The other key issue that needs to be discussed is the higher fraction of LAGBs and average grain size in the sample MDFed at a high temperature (177 °C) compared to the sample processed at RT. As the processing temperature increases and the flow localization decreases, the applied strain by shear bands and the number of dislocation cells decrease, leading to a higher average grain size [33]. On the other hand, MDF at high temperatures is accompanied by dynamic recovery that could lead to grain growth and larger average grain size. The formation of new grains in aluminum alloys is usually a result of LAGBs transformation into HAGBs which is controlled by the recovery rate. The transformation of LAGBs into HAGBs occurs due to the addition of mobile dislocations into the grain boundaries during plastic deformation. On the other hand, the annihilation of dislocations within the grains happens as a result of dynamic recovery at higher temperatures, and fewer dislocations will join the boundaries consequently. Thus, the increase of the misorientaion angle at higher temperatures is more difficult to achieve relative to room temperature. Therefore, the processed sample at 177 °C has a higher fraction of LAGBs [34,35,36,37].

### 3.2. Mechanical Properties

**Figure 7:**
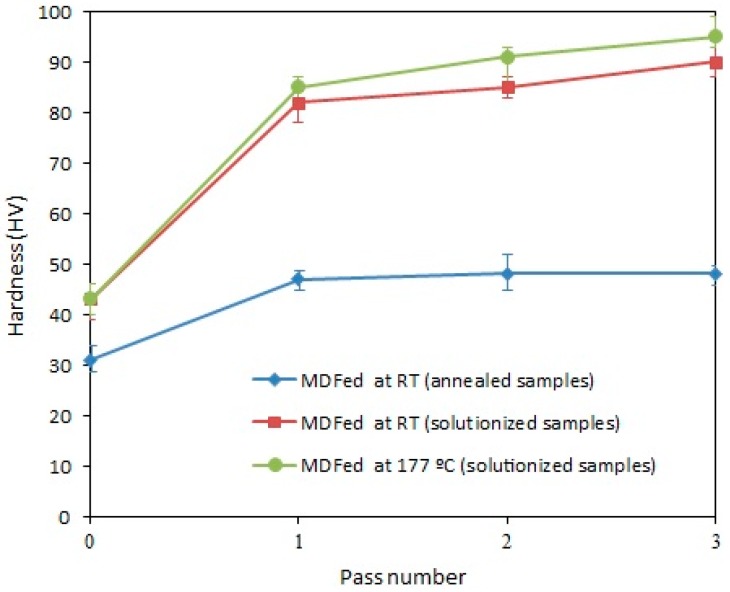
Hardness variations of the annealed and solution-treated samples of AA-6063 with respect to the MDF pass number.

Figure 7 presents the hardness of the annealed and solution-treated samples of AA-6063 alloy with respect to the pass numbers. The hardness of the annealed samples (31 HV) reached much high values after three passes of MFD (48 HV) representing a 50% increase. The hardness of the solution-treated samples (43 HV) after three passes at RT and 177 °C showed much higher increases of 109 and 121% and reached 90 and 95 HV, respectively. In all cases, the increase in the hardness value is mostly achieved after the first pass, while the hardness of the annealed samples remaining almost constant after the second pass and the solution-treated samples display a slight increase after the second and third passes. The samples processed at 177 °C have a higher hardness than the RT samples in all passes.

**Figure 8:**
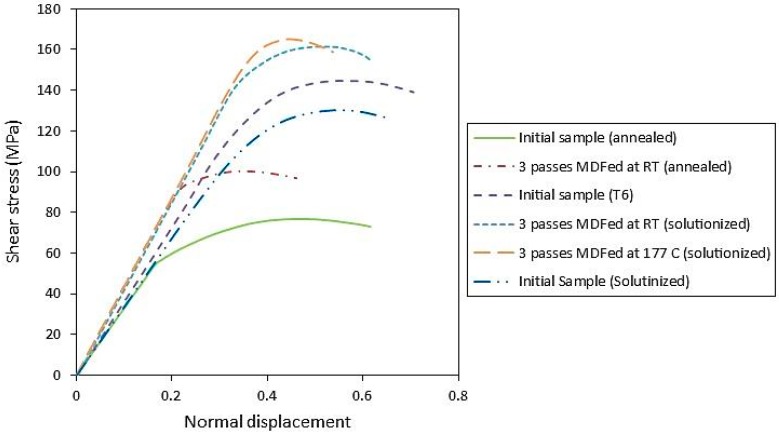
Shear stress versus normal displacement for annealed, solution-treated, and T6 samples of AA-6063 alloy.

The shear stress versus normal displacement of the annealed and solution-treated samples of AA-6063 is shown in Figure 8 which also includes a heat treated sample under T6 conditions to make a comparison between the MDFed and T6-treated samples. The extracted information from these graphs including shear yield strength ($\tau_{Y}$), ultimate shear strength ($\tau_{\mathrm{UTS}}$), hardening ration ($\tau_{\mathrm{UTS}}$/$\tau_{Y}$) are summarized in Table 1. The shear yield stress and ultimate shear stress of the annealed sample after three passes of MDF increased by 56 and 30%, respectively. Shear punch test also resulted in a 72% rise in the shear yield stress and a 24% increase in the ultimate shear stress for the solution-treated samples processed at RT. These values are even higher for the samples processed at 177 °C as the shear yield and ultimate shear stress were reported by 83% and 27% higher than the initial values, respectively.

The achieved improvement in the mechanical properties of the annealed and solution-treated samples of AA-6063 is closely related to the increase of LAGBs, grain refinement and significant increase in the density of dislocations. The Hall–Petch equation also proves that the yield stress and other mechanical properties increase accordingly as the average grain size of all samples decreases. The relationship between yield stress and average grain size (Hall–Petch equation) is as follows [38]:

(5) σY=σo+kyd−12

$\sigma_{Y}$, *σ_o_*, *k_y_*, and *d* are the yield strength, a materials constant for the starting stress for dislocation movement, strengthening coefficient, and average grain size, respectively. Many of the previous researches also confirm the validity of Hall–Petch equation for the metals and alloys processed by SPD methods with ultrafine grains ranging from 1 µm to 100 nm [39,40,41,42,43,44].

Another basic strengthening mechanism in metals and alloys is strengthening through dislocations. SPD methods increase the density of dislocations, and as a result, the mobile dislocations interact with themselves and impede their own motion. Thus, the increase of dislocation density leads to an improvement in the mechanical properties. According to the Taylor model, the rise of yield strength due to the high density of dislocations during deformation is calculated as follows [45,46,47,48,49,50]:

(6) σd=σo+MαGbdρ12,

*ρ*, α, M, G, and b represent the density of dislocations, a material constant, Taylor factor (three for polycrystalline metals), shear modulus (26 GPa for aluminum), and the magnitude of Burgers vector (0.286 nm for aluminum), respectively.

The solution-treated samples of AA-6063 processed by three passes at both temperatures indicated better mechanical properties compared to the T6-treated sample. This can be attributed to the simultaneous effect of grain refinement, work hardening and dynamic aging process. As the precipitation rate accelerates during mechanical working, dynamic aging can occur. The MDF process presumably provides some favorable nucleation sites through increasing the density of dislocations and hastening the diffusion process especially at higher temperatures which leads to a higher precipitation rate. The accumulation of shear bands as high-energy and favorable nucleation sites during the MDF process is also another factor resulting in a higher precipitation rate [51,52,53]. Since the aging process is affected by temperature and time, processing the solution-treated samples of AA-6063 by three passes at 177 °C brings about better mechanical properties compared to the three-pass MDFed samples at RT as higher temperature accelerates the precipitation and aging process. Hence irrespective of the higher average grain size and less work hardening at 177 °C compared to the processed sample at RT, age hardening is possibly the main reason behind the more desirable mechanical properties at high temperature.

**Figure 9:**
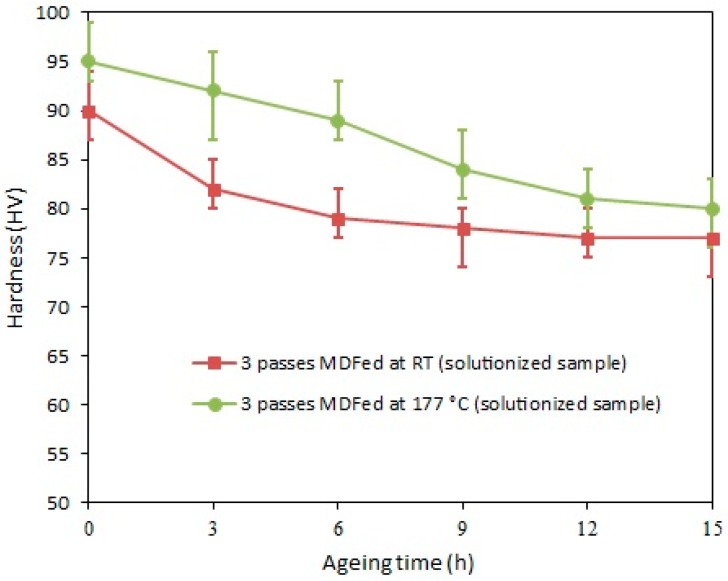
Hardness- aging time curves of the solution-treated samples of AA-6063 MDFed for three passes at room temperature and 177 °C.

To investigate the extent of aging achieved during the MDF process at RT and 177 °C, the three-pass MDFed samples were aged at T6 temperature (177 °C). Figure 9 demonstrates the changes in the hardness value with respect to the aging time. The obtained results showed that the hardness value of the three-pass MDFed sample at RT after being aged for 3, 6, 9, 12, and 15 h has fallen from 90 HV to 82, 79, 78, 77, and 77 HV, respectively. On the other hand, the hardness value of the three-pass MDFed sample at 177 °C gradually decreased from 95 HV to 92, 89, 84, 81 and 80 HV after 3, 6, 9, 12, and 15 h of aging, respectively. The thermal recovery and possible grain growth are the prevailing factors in contributing to the decrease of the mechanical properties including hardness during post-MDF aging. Additionally, a significant part of the aging process also occurs dynamically during the MDF process, especially at high temperature. Hence the maximum hardness value is possibly achieved during forging as a result of dynamic aging, and post-MDF aging just makes the precipitates grow and lose coherency, leading to lower hardness values [54].

### 3.3. Electrical Properties

**Figure 10:**
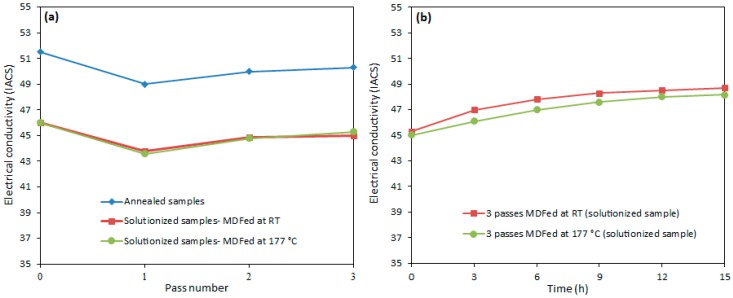
Electrical conductivity changes of the annealed and solution-treated samples of AA-6063 with respect to: (**a**) the number of MDF passes and (**b**) aging time.

Since the AA-6063 alloy has some electrical applications in electrical substations and electrical components due to its proper electrical conductivity, it is interesting to study the effect of deformation processes such as MDF on electrical properties of this alloy. Figure 10 represents the evolution of the electrical conductivity for the annealed and solution-treated samples of AA-6063 with respect to the number of MDF passes. Table 2 also contains a summary of the obtained results.

The electrical conductivity of both AA-6063 conditions does not vary significantly with MDF. The obtained results showed that the electrical conductivities decrease slightly under both conditions at the first pass suggesting that a considerable increase in plastic deformation is known to cause a disturbance in the movement of free electrons through the crystal structure as dislocations have a dispersive effect on electrons leading to a lower electrical conductivity [55,56]. However, as the number of passes increases, more dislocations join into LAGBs and consequently, the density of dislocations decreases and the free movement route of electrons lengthens and electrical conductivity slightly increases. Thus, the slight increment of conductivity in the second and third passes can be ascribed to a decrease in the density of dislocations as LAGBs transform into HAGBs. It can be concluded that although the impact of MDF process on the grain refinement, as well as enhancement of surface density of LAGHs and HAGBs, is significant (Figure 2, Figure 3, Figure 4, Figure 5 and Figure 6), it has been almost negligible on the electrical conductivity as grain boundaries are not serious obstacles to the movement of free electrons. It is the dislocations’ interaction with grain boundaries (LAGBs and HAGBs) that results in a huge improvement in the mechanical properties. 

Electrical conductivity in metals is closely correlated with the microstructure and is highly affected by the structural defects including vacancies, dislocations, grain boundaries, impurities, etc. Hence, improving the strength by adding alloying elements, work hardening and precipitation hardening of metals is typically associated with a decrease in conductivity [15,57,58]. Alloying is one of the most common ways to increase the strength of metals as was mentioned earlier. However, it is associated with some downsides, such as a fall in electrical conductivity. So it would be very advantageous to find a way to increase the strength with no tangible fall in conductivity. In the current investigation, the MDF process increased the mechanical properties notably without an appreciable decrease in the electrical conductivity [15,57,58]. Therefore, it can be concluded that grain refinement by SPD processes is a useful method to improve the mechanical properties without having a notable decrease in the electrical conductivity. In other words, a better combination of mechanical strength and electrical conductivity can be obtained by processing the AA-6063 alloy by the MDF process compared to the commercial Al-Mg-Si alloy processed via conventional thermo-mechanical treatments.

Furthermore, the effect of the aging time on the electrical conductivity of the solution-treated samples of AA-6063 MDFed for three passes was investigated. According to Figure 10b, there has been some improvement in the electrical conductivity of the samples with an increase of aging time. This happened as a result of the dynamic recovery mechanism and the formation of more precipitates. The depletion of alloying elements as a result of precipitates formation reduces the negative effect imposed by the alloying elements’ tension field on the movement of free electrons which leads to an increase in conductivity. It should be noted that the solute atoms dissolved in the matrix are much more effective in scattering electrons and consequently a reduction in electrical conductivity compared to other obstacles [15,59,60].

### 3.4. Texture Analysis

**Figure 11:**
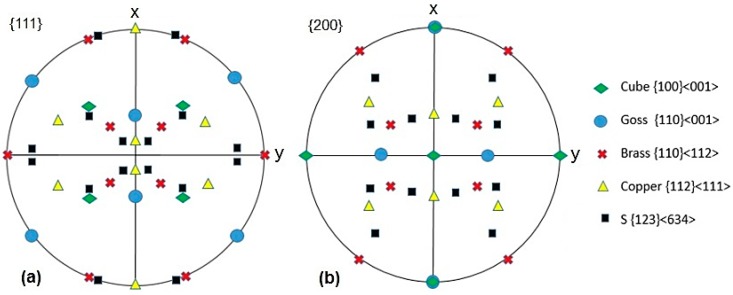
The typical orientation and ideal texture of a face centered cubic material in: (**a**) {111} and (**b**) {200} pole figures.

Texture evolution of the annealed and solution-treated samples of AA-6063 MDFed at both temperatures was also studied. In this regard, the typical orientation and ideal texture of a face centered cubic material are shown in Figure 11 in {111} and {200} pole figures. Copper {112} <111>, brass {011} <211>, S {123} <634>, cube {001} <100>, and Goss {011} <100> are the components included in Figure 11 [31].

**Figure 12:**
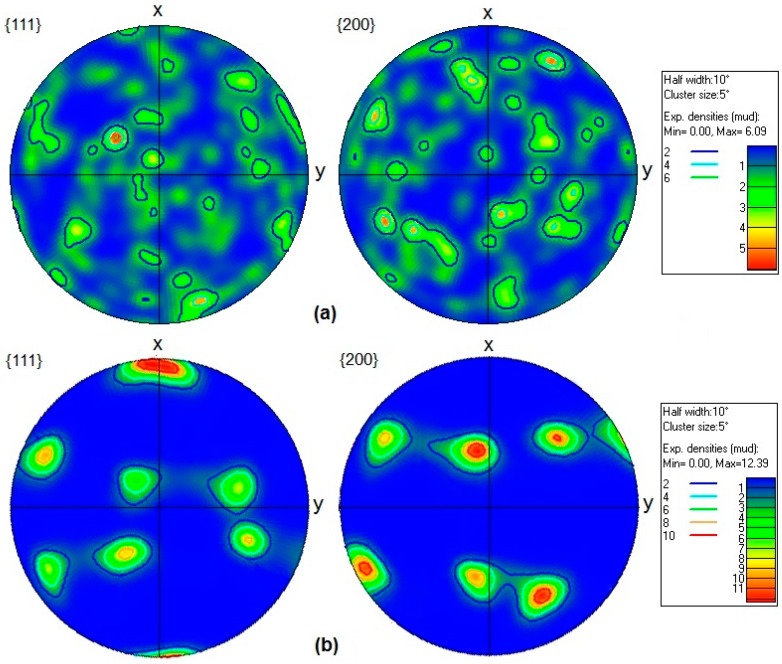
{111} and {200} pole figures of the annealed AA-6063 alloy: (**a**) before and (**b**) after three passes of MDF and their related texture intensity indicators.

**Figure 13:**
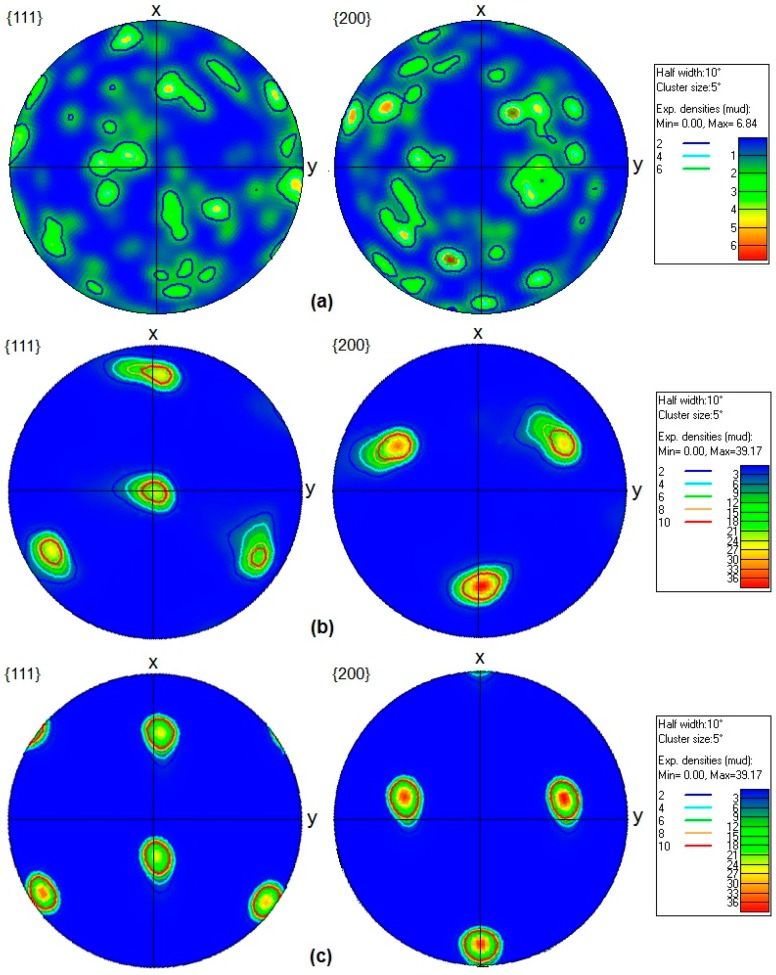
{111} and {200} pole figures of the solution-treated AA-6063 alloy: (**a**) before (**b**) and (**c**) after three passes of MDF at room temperature and 177 °C, respectively, and their related texture intensity indicators.

{111} and {200} pole figures of the annealed and solution-treated samples of AA-6063 before and after three passes of MDF and their related texture intensity indicators are depicted in Figure 12 and Figure 13. Both the annealed and solution-treated samples of AA-6063 show almost no texture before the MDF process. Figure 12 shows the formation of a textured fiber <111> including copper texture components in the annealed sample when is processed up to three passes of MDF. The fiber texture is displayed by more intense texture intensity on the northern and southern poles. On the other hand, Goss texture components were mainly formed in the solution-treated samples subjected to three passes of MDF at both temperatures. The comparison of texture intensity indicators in Figure 12 and Figure 13 reveals that strong texture forms during MDF process at both temperatures.

In FCC metals with a medium to high stacking fault energy (SFE), grains have a tendency to form a copper-type texture as the plastic deformation increases [61]. The presence of high amount of precipitates in the annealed sample is probably the reason of formation different texture during MDF in annealed samples compared to solution-treated samples. It’s likely that the aforementioned precipitates hinder the free rotation of grains. The formation of different textures in differently heat-treated aluminum alloy after MDF process has been reported before as well. It was ascribed to the distinct microchemistry of the matrices and the high density of deformation bands in the solution treated samples since the higher solute content retards dynamic recovery as reported for MDFed over-aged and solution treated Al-Cu-Mg alloy [62]. 

## 4. Conclusions

The effect of the MDF process on the microstructure, texture, mechanical and electrical properties of both annealed and solution-treated samples of AA-6063 was investigated. According to the results obtained, the main conclusions of this investigation are as follows:

(1) EBSD analyses showed the average grain size of both annealed and solution-treated samples has decreased noticeably during the MDF process. For instance, the average grain size of the solution-treated sample MDFed at room temperature reduced from 200 µm for the initial sample to 1 µm after three passes. In addition to grain refinement, all samples also indicated a considerable increase in the density of LAGBs and most of the grains are elongated along the deformation bands.

(2) The hardness of both annealed and solution-treated samples demonstrated an appreciable increment after being processed by MDF. For instance, the hardness value of the solution-treated samples MDFed at RT and 177 °C experienced more than 100% increase compared to the initial microstructure. The increase in the hardness value is much more noticeable after the first pass and become less efficient for the next passes. Development of UFG structure, work hardening (as a result of the increased density of dislocations) and the dynamic aging process are the main factors behind the improvement of hardness.

(3) Shear punch testing results of the annealed sample after three passes of MDF showed 56 and 30% increase in shear yield strength and ultimate shear strength, respectively. The solution-treated samples processed at RT and 177 °C also showed 72 and 83% rise in the yield strength and 24 and 27% increases in the ultimate shear strength, respectively. These improvements could also be attributed to the above-mentioned reasons for the hardness increase.

(4) Post-MDF aging of three-pass MDFed AA-6063 samples at RT and 177 °C led to a reduction in the mechanical properties. Because the maximum hardness value was achieved during the MDF process as a result of dynamic aging and post-MDF aging just makes the precipitates grow and lose coherency.

(5) Effect of the MDF process on the electrical conductivity of both solution-treated and annealed samples of AA-6063 was found to be negligible. The electrical conductivity decreases negligibly after the first pass and shows a slight increase in the second and the third passes.

(6) Texture analyses revealed that a relatively strong copper-type texture component was formed in annealed samples of AA-6063 alloy MDFed up to three passes, while, a relatively strong Goss texture component was formed in solution-treated samples after three passes of the MDF process at both room and 177 °C temperatures. The reason for this difference lies in the different amount of precipitates and distinct microchemistry of the matrices in the annealed and solution-treated samples.

## Figures and Tables

**Table 1:** Mechanical testing results for the annealed, solution-treated, and T6 samples of AA-6063.

Sample	Shear Yield Stress (MPa)	Ultimate Shear Stress (MPa)	$\tau_{YUTS}/\tau_{Y}$	Hardness (HV)
Initial sample (annealed)	52	77	1.6	31
3 passes MDFed (annealed)	75	100	1.3	48
Initial sample (solution-treated)	79	130	1.6	43
Heat treated sample (T6)	101	145	1.4	76
3 passes MDFed at RT (solution-treated)	136	162	1.2	90
3 passes MDFed at 177 °C (solution-treated)	145	165	1.1	95

**Table 2:** Electrical conductivity measurement results (annealed and solution-treated samples of AA-6063).

Samples	Initial Sample (IACS)	First Pass (IACS)	Second Pass (IACS)	Third Pass (IACS)
Annealed	51.5	49.0	50.0	50.3
Solution-treated at RT	46.0	43.8	44.9	45.0
Solution-treated at 177 °C	46.0	43.6	44.8	45.3
